# ﻿New synonymy among gall thrips of the Asian genus *Mesothrips*, with revision of species from China (Thysanoptera, Haplothripini)

**DOI:** 10.3897/zookeys.1196.118131

**Published:** 2024-03-22

**Authors:** Lihong Dang, Xiaoli Tong, Laurence A. Mound

**Affiliations:** 1 School of Bioscience and Engineering, Shaanxi University of Technology, Hanzhong, 723000, China Shaanxi University of Technology Hanzhong China; 2 Shaanxi Province Key Laboratory of Bioresources, Hanzhong, 723000, China Shaanxi Province Key Laboratory of Bioresources Hanzhong China; 3 Qinba Mountain Area Collaborative Innovation Center of Bioresources Comprehensive Development, Hanzhong, 723000, China Qinba Mountain Area Collaborative Innovation Center of Bioresources Comprehensive Development Hanzhong China; 4 Qinba State Key Laboratory of Biological Resources and Ecological Environment (Incubation), Hanzhong, 723000, China Qinba State Key Laboratory of Biological Resources and Ecological Environment (Incubation) Hanzhong China; 5 College of Plant Protection, South China Agricultural University, Guangzhou 510642, Guangdong Province, China South China Agricultural University Guangzhou China; 6 Australian National Insect Collection CSIRO, PO Box 1700, Canberra, ACT 2601, Australia Australian National Insect Collection CSIRO Canberra Australia

**Keywords:** Key, *M.jianfengi*, *M.longistylus*, *M.vernicia*, new species

## Abstract

Historical, nomenclatural, technical, and biological problems associated with the 42 species of *Mesothrips* are discussed. Type specimens have been re-examined of 14 of the 25 species that were described prior to 1930 and remain known only from imperfectly slide-mounted specimens. As a result, seven new synonyms are recognised. From China, six species of *Mesothrips* have been listed, but the records of *M.alluaudi* and *M.manii* are rejected, and three new species are described: *M.jianfengi***sp. nov.**, *M.longistylus***sp. nov.**, and *M.vernicia***sp. nov.** These three species are divergent from other members of *Mesothrips* in lacking a prominent fore tarsal tooth and may indicate a possible generic relationship to the flower-living species in the Asian genus *Dolichothrips*. An illustrated key is provided to the seven *Mesothrips* species now known from China.

## ﻿Introduction

**Figure 1.**
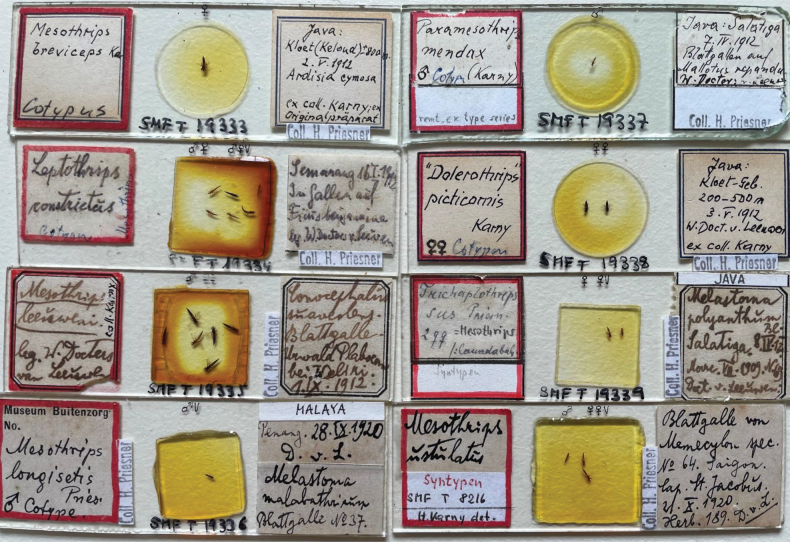
Type specimens of eight species that have been referred to the genus *Mesothrips*.

The genus *Mesothrips* Zimmermann, 1900 continues to comprise one of the most misunderstood groups of leaf-feeding species in the subfamily Phlaeothripinae. The 42 species listed under this genus name are all presumed to be gall-living, with some of them almost certainly gall-inducing (ThripsWiki 2023). Publications referring to these thrips involve a series of problems. At least 25 of the species listed in the genus were described prior to 1930. They were based on specimens that were not mounted to modern standards and are difficult to study and compare (Fig. 1). Subsequent recordings of these named species by other authors are commonly based solely on published data, not on study of type specimens, and there are no studies on the life-history, host specificity, or dispersive activity of these species. As a result, much of the published taxonomy for this genus requires further verification, based on good field samples rather than just re-examination of the 100-year-old type specimens.

The problems began with the original description of the genus and the five new species that Zimmermann included and described. The first of these, *Mesothripsuzeli* Zimmermann, 1900 was also placed by Zimmermann as the only species of his second new genus, *Gynaikothrips* Zimmermann, 1900. In pointing this out, Priesner (1929a) indicated that three of the other five listed species should also probably be placed in *Gynaikothrips*, and so he recognised *Mesothripsjordani* Zimmermann, 1900, the fourth of Zimmermann’s species, as the type species of *Mesothrips*. Priesner further listed 16 species that he considered likely to be members of this genus, of which *M.australiae* Hood, 1918 is now placed as a synonym of *M.jordani*. However, the 16 species did not include all the names available in this genus at the time Priesner was writing, with three species by Karny not mentioned: *M.armatus* Karny, 1913, *M.picticornis* Karny, 1913, and *M.lividicornis* Karny, 1923, nor *M.alluaudi* Vuillet, 1914 from Madagascar and *M.sus* (Priesner, 1921) from Java.

Ananthakrishnan (1976) is the only author to attempt an extensive revision of the members of *Mesothrips*, and he provided a key to 28 of the 37 species known at that date. The first couplet of the key appears to be clear; eight species have the fore wings either clear or with light shading around the margins distally, whereas all the other species have fore wings more or less shaded. But interpretation within the key of the first of these choices seems inconsistent, because of the eight species treated as having clear fore wings two actually have the distal half of the wing shaded, *M.constrictus* (Karny, 1912) and *M.leeuweni* Karny, 1913. A similar problem arises with decisions concerning wing colour in couplets 9 and 10. Certainly the fore wings of some species are fully shaded, such as *M.breviceps* Karny, 1913, but the fore wings of *M.jordani* are shaded only on the distal half, the fore wing basal half being pale and with no longitudinal markings. Unfortunately, in slide mounted specimens that have been cleared for critical study the fore wings may have lost much of their colour, and wing colour may vary between individuals in a population, in relation to maturity and body size. Head length has also been used to distinguish species in this genus, although this length is interpreted here as being variable within and between populations of *M.jordani*. Similarly, the relative lengths of the pronotal epimeral and postero-angular setae have been used to distinguish species, but these setae are also more variable than some authors have considered. The key to nine *Mesothrips* species from India (Ananthakrishnan and Sen 1980) includes similar problematic decisions. As a result, in the present study of *Mesothrips* from China, some species are newly placed into synonymy, based on examination of the original specimens.

Two further authors have produced identification keys to species of this genus from particular areas. Reyes (1994) recorded five species from the Philippines, although the first species in that key, *M.ignotus* Reyes, 1994, is now recognised as a member of the genus *Adelphothrips* Priesner, 1953 (ThripsWiki 2023). Moreover, the statement in the key that the compound eyes of *M.leeuweni* are prolonged ventrally is not correct. Some syntypes of that species certainly have the pigment of the eyes migrated posteriorly and somewhat distant from the eye structure, but the posterior margin of the eye itself is visible and the eye is slightly shorter ventrally than dorsally. Okajima (2006) provided a clear generic diagnosis together with a key to the two species known from Japan, *M.claripennis* Moulton, 1928 and *M.jordani*. The first of these has the fore wings uniformly pale and lives on *Ardisia* [Primulaceae], whereas the second has the wings uniformly shaded on the distal half and lives on *Ficus* [Moraceae].

Interpreting the taxonomic significance of structural variation between populations of gall thrips involves problems. Previous authors have commonly stressed slight structural differences, considering that these distinguish species, although such hypotheses remain untested. An alternative hypothesis is equally valid, that small structural differences are intra-specific and reflect in gall-thrips populations a founder effect. This second hypothesis assumes that a population within a single gall is usually the progeny of a single, once-mated, female, and hence shows limited structural variation. Similarly, in the absence of evidence of dispersive activity by *Mesothrips* species, it can be assumed that the members of a population on a single tree are likely to be closely related and more similar to each other than to individuals from more distant populations. Earlier taxonomic work appears to have assumed that speciation in this genus has sometimes occurred on single plant species, such as the common *Ficusbenjamina*. However, we suggest that speciation within the genus *Mesothrips* is related to differences between host plant species, although the available host data to support this suggestion remains inadequate. This dichotomy in hypotheses is involved here in interpreting variation among individuals of what we consider to be a single species, *M.jordani*.

### ﻿Notes on species diversity of *Mesothrips*

It is currently not possible to produce a revision of the species listed in this genus, as the type specimens are widely dispersed amongst various collections, and some are not available for study. For production of this account of *Mesothrips* species from China, type specimens (Fig. 1) of 12 nominal species were borrowed from Senckenberg Museum during a visit by LAM in 2023, and *M.pyctes* Karny, 1916 and *M.elaeocarpi* Ananthakrishnan, 1976 were previously studied by LHD in 2012 (Dang et al. 2014).

*Mesothripsbreviceps* Karny (1913: 69) is the only species available for study with uniformly shaded fore wings. One syntype from Java on *Ardisiacymosa* [Primulaceae] has been studied (from SMF). Several species have been studied with the fore wings uniformly pale or with only the extreme margins slightly shaded. One of these, *M.longisetis* Priesner, 1929, has exceptionally long pronotal and postocular setae, and is known from *Melastomamalabathricum* [Melastomataceae] in Malaya. However, *Mesothripssus* was described from two females as the only member of a new genus *Trichaplothrips* Priesner, 1921. These specimens were taken at Salatiga in central Java from *Melastomapolyanthemum*, and these two plant names are now considered to refer to the same plant species, *Melastomaaffine*. A syntype male of *M.longisetis* has been studied and compared to the two syntype females of *M.sus* (Fig. 7) and these are here considered to represent the same species. *Mesothripslongisetis* is therefore considered a new synonym of *M.sus*.

*Mesothripsschouteniae* Priesner, 1929 has fore wings pale with margins slightly shaded and antennae largely yellow, but with pronotal setae less elongate than *M.sus*; it was described from Java on *Schouteniaovata* [Malvaceae]. Also with pale fore wings is *M.vitripennis* Karny, 1922 (Fig. 19) from Vietnam on *Aporosaleptostachya* [Phyllanthaceae], and *M.moundi* Ananthakrishnan, 1976 (Figs 6, 11) from Hong Kong on *Bischofiatrifoliata* [Phyllanthaceae] is considered below as a new synonym of *M.vitripennis*. Similarly, *M.elaeocarpi* (Figs 27, 29) is here considered a new synonym of *M.vitripennis*.

*Mesothripsmendax* (Karny, 1912) also has pale fore wings but is distinctive in having slender fore femora and the mouth cone pointed; this species was taken in central Java in leaf galls on *Mallotusrepandus* [Euphorbiaceae]. Another species with similar fore wings is *M.ustulatus* Karny, 1912 from a leaf gall on *Memecylon* [Melastomataceae] in Vietnam, but the two available syntypes are too poorly preserved for serious comparisons with other species.

The published descriptions of three species from India, *M.extensivus* Ananthakrishnan & Jagadish, 1969, *M.acutus* Muraleedharan & Sen, 1981 and *M.ambasensis* Muraleedharan & Sen, 1981, also state that the fore wings are pale; the first was described from galls on *Anogeissus* [Combretaceae], but the other two merely from galls with no host plant record. In contrast, *M.manii* Ananthakrishnan, 1972 was described from southern India as having fore wings with a yellowish tinge, based on a long series of both sexes from leaf galls on *Santalumalbum* [Santalaceae]. The species *M.pyctes* (Figs 26, 28, 30, 31) from Java on *Eugenia* sp. [Myrtaceae] was described as having the fore wings “schwach graulich” (weakly greyish), and the type specimens of this species need to be compared to original specimens of *M.manii* to determine if these represent different species. *Mesothripsmanii* has been recorded from China twice (Zhang 1984; Zhang et al. 1999), but the specimens on which these records were based, from Hainan and Fujian respectively, have now been re-examined and are here identified as *M.jordani* and *Bamboosiella* sp.

*Mesothripsjordani* is here considered to be widespread on the leaves of *Ficusbenjamina* [Moraceae], and this species is interpreted here as having a characteristic fore wing colour - uniformly shaded on the distal half with the basal half pale and lacking any dark median line (Fig. 18). Three species are listed below as new synonyms of *M.jordani*. However, *M.apatelus* Karny, 1926 from India, known only from descriptions and the key by Ananthakrishnan and Sen (1980), is possibly a further synonym of this species.

*Mesothripsleeuweni* Karny has fore wings that are rather similar to those of *M.jordani* but with a more obvious median dark line on the basal half of the fore wing, also the antennal segments are more extensively pale than is usual in *M.jordani*. Karny recorded *M.leeuweni* at more than one site in Java in leaf galls on *Poikilospermumsuaveolens* [Urticaceae], although he used the generic name *Conocephalus* that is now considered a synonym of *Poikilospermum*.

*Mesothripspicticornis* Karny, 1913 also has the distal half of the fore wing shaded but is distinctive in having antennal segments III–V extensively blackish brown with the base of each segment pale. Karny described this species from Java as taken in leaf galls on *Vitispapillosa* [Vitaceae].

Moulton (1942) described *M.guamensis* Moulton, 1942 from a single male, and *M.swezeyi* Moulton, 1942 from a single female, both specimens having been taken together under the bark of a tree in Guam. These specimens have now been re-examined and have broad maxillary stylets arranged in a V-shape and retracted almost to the postocular setae. Moreover, all the antennal segments are dark brown. These specimens are here recognised as members of the Idolothripinae genus *Ethirothrips* Karny, 1925. Neither specimen has been cleared before slide mounting and they are thus difficult to study. However, the two species are here both considered to be new synonyms of the widespread species *Ethirothripsstenomelas* (Walker, 1859), of which an excellent modern description is available (Okajima and Masumoto 2023).

### ﻿Generic relationships

The genus *Mesothrips* is a member of the tribe Haplothripini Priesner, as redefined by Mound and Minaei (2007) from the *Haplothrips*-lineage of Mound and Marullo (1996). The species in this tribe have the fore wings with a median constriction, although this varies from being almost a pouch to being scarcely visible. The prosternum has a pair of basantral sclerites, and the metathoracic sternopleural sutures are not developed. In the males, tergite IX setae S2 are shorter than setae S1, and sternite VIII lacks a pore plate. Species of *Haplothrips* Amyot & Serville, 1843 (with very few exceptions) have either one or two sense cones on antennal segment III, whereas all species previously assigned to *Mesothrips* have three sense cones on this segment. The type species of *Mesothrips* has the head distinctly constricted at the base, but in other members of the genus this constriction is only weakly developed. As a result, these species share character states with *Dolichothrips* Karny, 1912, an Asian genus in which the species are not gall-living but are commonly found in flowers, often of the genera *Macaranga* and *Mallotus* (Mound and Okajima 2015). The distinction between these two genera becomes more confused by the description below of three new species in the genus *Mesothrips*, all of which lack a prominent tooth on the fore tarsus, and one of them has only two sense cones on the third antennal segment. These three species share several character states and are presumably closely related to each other. However, their generic relationships are far from clear, and they are described here in the genus *Mesothrips* primarily because of the additional sigmoid setae on the tergites. It is possible that the forests of southwestern China have a previously unknown diversity of leaf-feeding Phlaeothripinae that will require future revisions of the generic classifications.

## ﻿Materials and methods

The descriptions and drawings were produced from slide-mounted specimens with a Nikon Eclipse 80i microscope. Images were prepared with a Leica DM2500 using DIC illumination, and processed with Automontage and Adobe Photoshop v.7.0. The abbreviations used for the pronotal setae are as follows: **am** – anteromarginal, **aa** – anteroangular, **ml** – midlateral, **epim**– epimeral, **pa** – posteroangular; **CPS** – campaniform sensilla. The unit of measurement in this study is the micrometre (μm). Most specimens studied here are available in the School of Bioscience and Engineering, Shaanxi University of Technology (**SNUT**), Hanzhong, China, the Australian National Insect Collection (**ANIC**), CSIRO, Canberra, Australia, and South China Agricultural University (**SCAU**). Further slides were studied on loan from the Senckenberg Museum, Frankfurt (**SMF**).

## ﻿Taxonomy

### ﻿ Mesothrips

Taxon classificationAnimaliaThysanopteraPhlaeothripidae

Zimmermann

BB60D02D-47C9-52B3-AF0B-AEDA9E1B8A48

Mesothrips Zimmermann, 1900: 12. Type species Mesothripsjordani Zimmermann, 1900, by subsequent designation of Priesner 1929a: 452.

#### Note.

From China six species have been recorded in this genus (Dang et al. 2014), but the records of *M.alluaudi* and *M.manii* are here rejected. *Mesothripsalluaudi* was recorded from China by Moulton (1928b: 318), based on a single female collected from *Machilus* sp. at Taihoku, Taiwan by R. Takahashi on 27 June 1927. Subsequent authors in China have repeated this record, but apparently without checking the original Moulton paper. The specimen from Taiwan was identified by Moulton based only on Vuillet’s original description of two specimens taken in Madagascar, and that original description is insufficient to place *M.alluaudi* with confidence into any genus. Moreover, it states that only two sense cones are present on the third antennal segment, in contrast to the three on *Mesothrips* species. The identity of the specimen from Taiwan that Moulton identified as this species remains unknown. *Mesothripsmanii* was based on a holotype female taken with over 100 adults of both sexes in leaf galls on *Santalumalbum* in Tamil Nadu, southern India. The original description states that the body length of females was 1718–2215 μm, and the head length 181–215 μm, with the fore wings having a yellowish tinge, and tergite IX setae S1 slightly shorter than the tube. Zhang (1984: 17) recorded *M.manii* from China on Hainan Island and subsequently Zhang et al. (1999) recorded this species from Fujian Province. We have now re-examined these specimens; the four males from Hainan are here recognised as *M.jordani* and from Fujian the single female is considered a species of the genus *Bamboosiella*.

#### Generic diagnosis.

**Figures 2–7.**
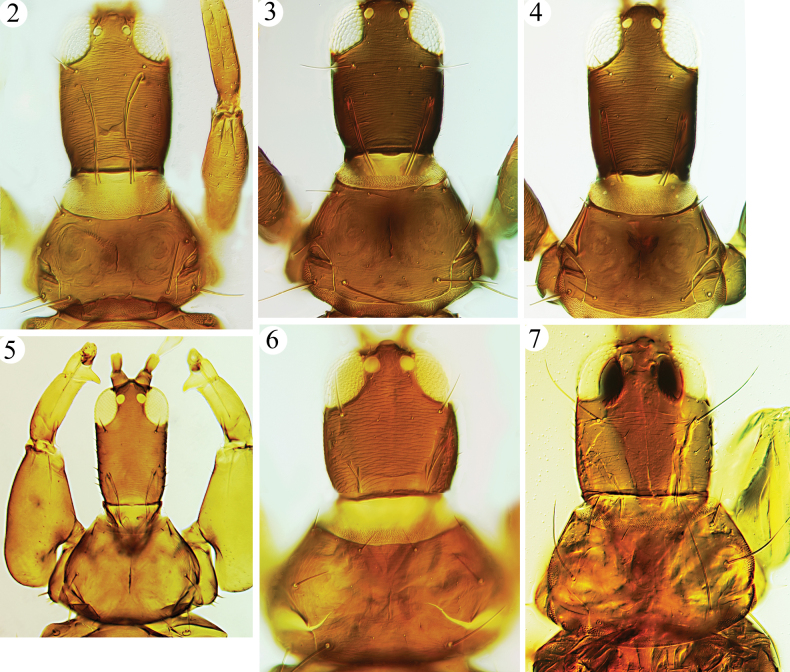
Head and pronotum **2***M.longistylus* sp. nov. **3***M.jianfengi* sp. nov. **4***M.vernicia* sp. nov. **5***M.jordani***6***M.vitripennis* (paratype female of *moundi*) **7***M.sus* (syntype female).

**Figures 8–13.**
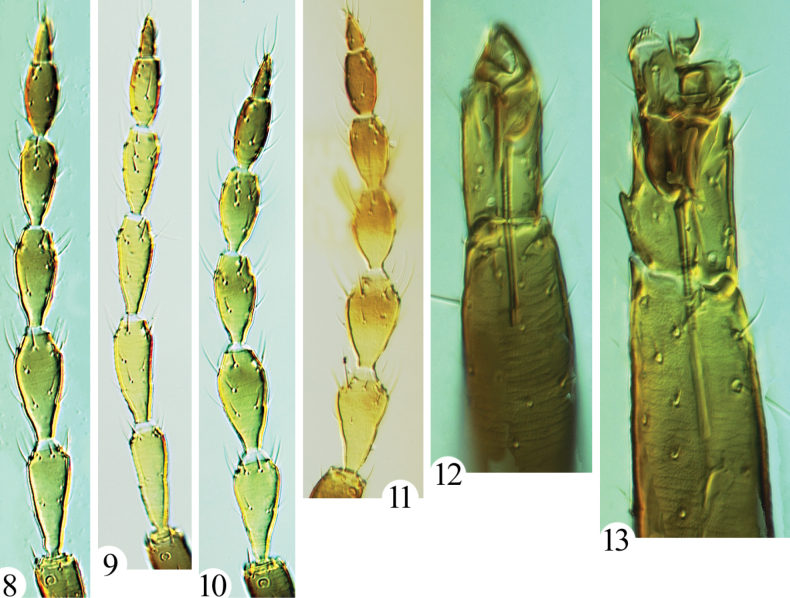
*Mesothrips* spp. Antennae (**8–11**) **8***M.longistylus* sp. nov. **9***M.vernicia* sp. nov. **10***M.jianfengi* sp. nov. **11***M.vitripennis* (paratype female of *moundi*); fore tarsi (**12–13**) **12***M.vernicia* sp. n. **13***M.jianfengi* sp. nov.

small to medium sized, dark, macropterous Phlaeothripinae-Haplothripini. Head usually longer than wide, cheeks sharply constricted at base (Figs 2–7); postocular setae developed; maxillary stylets rather short, usually not reaching postocular setae, V-shaped (rarely reaching postocular setae, and parallel medially); maxillary bridge present. Antennae 8-segmented (Figs 8–11, 31); segment III with three (rarely two) sense cones, IV with four major sense cones; VIII usually short. Pronotum well developed, with five pairs of major setae (Figs 2–7, 26, 27); notopleural sutures complete. Prosternal basantra present; ferna well developed; mesopresternum usually divided into two lateral triangular plates; metathoracic sternopleural sutures absent. Fore tarsal tooth usually large in both sexes (Fig. 5), sometimes absent or scarcely visible (Figs 12, 13). Fore wings constricted medially, with duplicated cilia (Figs 18, 19). Pelta triangular, usually with a pair of CPS (Fig. 16); tergites II–VII each with two pairs of sigmoid wing-retaining setae, also usually with a pair of accessory sigmoid setae anterior to first pair (Figs 17, 21, 22); tergite IX setae S1 and S2 long and pointed (Figs 28, 29). Male tergite IX setae S2 short (Figs 24, 25); sternite VIII without pore plate.

### ﻿Taxonomic account

#### ﻿Key to *Mesothrips* species from China

**Table:** 

1	Fore tarsal tooth absent or minute and visible only when tarsus rotated (Figs 12, 13)	**2**
–	Fore tarsal tooth present, usually large and pointed (Fig. 5)	**4**
2	Maxillary stylets parallel medially with transverse maxillary bridge, elongate and at full retraction extending to postocular setae (Fig. 2)	***M.longistylus* sp. nov.**
–	Maxillary stylets wide apart, arranged in V-shape, short and not reaching to postocular setae (Figs 3–6)	**3**
3.	Antennal segment III with only 2 sense cones (Fig. 9)	***M.vernicia* sp. nov.**
–	Antennal segment III with 3 sense cones (Fig. 10)	***M.jianfengi* sp. nov.**
4	Tergite IX setae S1 and S2 approx. as long as or longer than tube (Fig. 29)	**5**
–	Tergite IX setae shorter than tube (Fig. 28)	**6**
5	Fore wings distal 1/2 uniformly but sometimes weakly shaded, basal 1/2 pale without any dark longitudinal marks (Fig. 18) [on *Ficus*]	***M.jordani***
–	Fore wings uniformly pale (Fig. 19)	***M.vitripennis***
6	Anal setae slightly shorter than tube, ~ 0.9 × as long as tube (in Okajima 2006)	***M.claripennis***
–	Anal setae much shorter than tube, ~ 0.6 × as long as tube (Fig. 28)	***M.pyctes***

### ﻿ Mesothrips claripennis

Taxon classificationAnimaliaThysanopteraPhlaeothripidae

Moulton

5940FC6B-E610-5A83-857B-6C5B602BCF1B

Fig. 23

Mesothrips claripennis Moulton, 1928a: 315.

#### Material examined.

1♀, China, Yunnan, Lincang, Cangyuan, from grasses, 8.vi.2021 (**SNUT**); 1♂, Guangdong (**SCAU**); 1♂, Hainan, Zhanxian, 11.vi.1981 (**SCAU**); 1♀, Hainan, Danzhou, from gall of *Aporosadioica* [Phyllanthaceae], 2.xi.2014, Shulan Yang (**SCAU**); 1♀, Guangxi, Nanning, from *Ficus* sp. [Moraceae], 11.vii.1986, Weiqiu Zhang (**SCAU**); 1♂, Guangxi, Longzhou, 27.vii.1985, Weiqiu Zhang (**SCAU**).

#### Comments.

Described from China on a single female taken on an unknown plant by Takahashi, 30.xii.1926 at “Kannonzan, Formosa”, it was subsequently taken by that collector from other localities and recorded rolling the leaves of *Bladhiasieboldia* (Takahashi 1936). *Bladhia* is now recognised as a synonym of the Primulaceae genus, *Ardisia*, and Okajima (2006) recorded the thrips from leaf rolls on this plant on the Ryukyu Islands, Japan. This species has a smaller body (~ 2.5 mm) in comparison to most *Mesothrips*, and the fore wings are entirely pale. The specimens listed above are identified as *M.claripennis* from the description in Okajima (2006).

### ﻿ Mesothrips jianfengi sp. nov.

Taxon classificationAnimaliaThysanopteraPhlaeothripidae

D874254B-89AF-54BF-98DF-6339170643B6

https://zoobank.org/3166AB9E-F018-42B5-9E4A-DF97D03517A1

**Figures 14–25.**
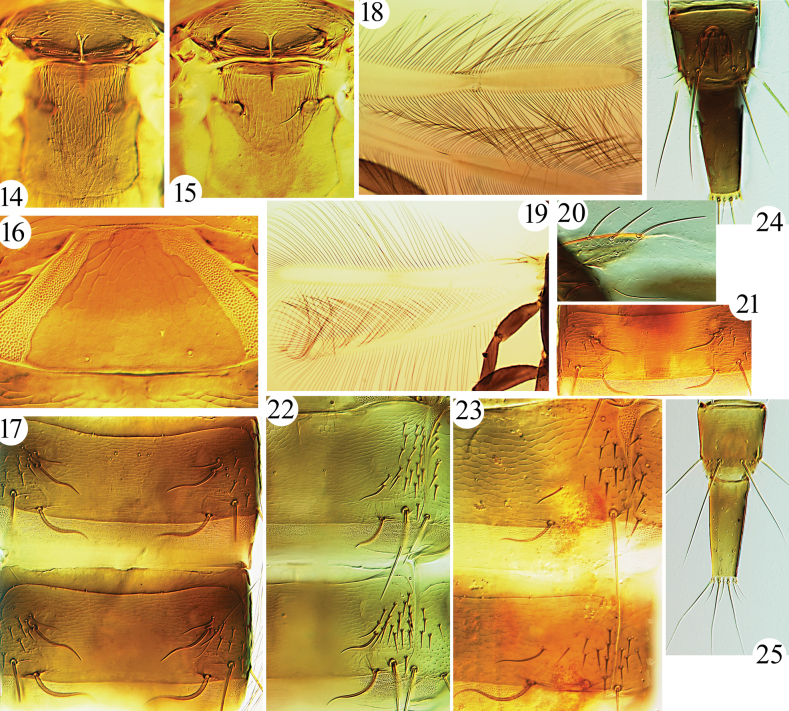
*Mesothrips* spp. Meso- and metanotum (**14–15**) **14***M.jianfengi* sp. nov. **15***M.longistylus* sp. nov.; *M.longistylus* sp. nov. (**16–17**) **16** pelta **17** tergites III–IV; fore wings (**18–20**) **18***M.jordani***19***M.vitripennis***20***M.longistylus* sp. nov., sub-basal setae of forewing; tergites II–III (**21–23**) **21***M.vernicia* sp. nov., tergite III **22***M.jordani***23***M.claripennis*; tergites IX–X (**24–25**) **24***M.longistylus* sp. nov. **25***M.vernicia* sp. nov.

Figs 3 , 10 , 13 , 14

#### Material examined.

***Holotype***, ♀, China, Xizang, Zhangmu, taken on leaves of unknown plant, viii.2013, Jianfeng Wang (**SNUT**); ***paratypes***, 2♀1♂, with same data as holotype (**SNUT**); 2♀1♂, Yunnan, Dali, on leaves of unknown plant, viii.2009, Lixin Su (**SNUT**).

#### Description.

**Holotype. *Female macroptera*.** Body brown; all femora and tibiae brown, fore tarsi clear yellow, mid and hind tarsi brownish, slightly lighter than tibiae; antennal segments I and II brown, III clear yellow, IV–VI yellow but shaded on apical half, VII and VIII brown (Fig. 10); major setae pale; fore wing slightly greyish.

***Head*.** Head ~ 1.3 × as long as wide (Fig. 3), constricted at base; postocular setae pointed or slightly blunt at apex, approx. as long as eyes (Fig. 3); eyes equal in length ventrally and dorsally; maxillary stylets V-shaped, retracted to median of head; mouth cone long, reaching to posterior margin of basantra. Antennal segments broad (Fig. 10), segment III ~ 2.1 × as long as apical width; III with three sense cones, IV with four major sense cones, VIII constricted at base.

***Thorax*.** Pronotum with five pairs of setae, slightly blunt at apex, am and aa approx. equal in length (Fig. 3), epim and pa longer and equal in length; surface almost smooth, with weak sculpture near margins. All legs slender, fore tarsi with minute and scarcely visible tooth (Fig. 13). Fore wing with three sub-basal setae arising in straight line, S1 and S2 equal in length, blunt at apex, S3 slightly longer, acute at apex, with eight duplicated cilia. Mesonotum transversely reticulate, lateral setae well developed, blunt at apex. Metanotum longitudinally reticulate, major setae slender and acute (Fig. 14). Mesopresternum with paired lateral triangles, metathoracic sternopleural sutures absent.

***Abdomen*.** Pelta broadly triangular, reticulate, with pair of CPS; tergites II–VII with two pairs of major wing-retaining setae, one pair of accessory sigmoid setae located anterior to first pair; tergite II with eight pairs of lateral setae; tergite IX setae S1 and S2 longer than tube, acute at apex, S3 approx. as long as tube, acute at apex; tube shorter than head, anal setae shorter than tube.

***Measurements*** (holotype female in μm). Body length 2820. Head length (maximum width) 250 (190); distance between maxillary stylets (across bridge) 115; eye length dorsally 90; postocular setae length 90; antennal segments I–VIII length (width): 45 (35), 50 (35), 75 (35), 75 (40), 65 (30), 60 (25), 55 (25), 35 (15); sense cone on III length 30. Pronotum length (width) 190 (270); am 40, aa 45, ml 60, epim 100, pa 100. Fore wing length 1160; sub-basal setae S1 90, S2 90, S3 110. Tergite IX setae S1 270, S2 240, S3 180; tube length 220, basal width 75, apical width 50; anal setae length 220.

***Male macroptera*.** Similar to female in colour and sculpture; fore tarsal tooth scarcely visible; fore wing with ~ 9 or 10 duplicated cilia; abdominal tergite IX setae S2 small and pointed; sternite VIII without pore plate.

***Measurements*** (paratype male in μm). Body length 2460. Head length (maximum width) 240 (175); eye length dorsally 85; postocular setae length 75. Pronotum length (width) 170 (230); am 35, aa 30, ml 40, epim 80, pa 85. Tergite IX setae S1 205, S2 25, S3 220; tube length 190; anal setae length 190.

#### Etymology.

The species epithet refers to one of the collectors, Jianfeng Wang (Shenyang University).

#### Comments.

This species is closely related to *M.longistylus* sp. nov., but it can be distinguished by the short V-shaped maxillary stylets (Fig. 3). The third new species described here, *M.vernicia* sp. nov., also has short stylets (Fig. 4), but *M.jianfengi* sp. nov. can be differentiated in antennal segment III with 3 sense cones (Fig. 10).

### ﻿ Mesothrips jordani

Taxon classificationAnimaliaThysanopteraPhlaeothripidae

Zimmermann

4A9385F7-5A07-513B-BA19-B0FDB1A3E300

Figs 5 , 18 , 22

Mesothrips jordani Zimmermann, 1900: 16.
Leptothrips angusticollis Karny, 1915: 88. Syn. nov.
Mesothrips annamensis Priesner, 1929b: 215. Syn. nov.
Leptothrips constrictus Karny, 1912: 150. Syn. nov.

#### Material examined.

1♀, China, Shaanxi, Hanzhong, 10.iv.2016, Kan Liu and Hui Lin (**SNUT**); 1♀, Fujian, Xiamen, from gall of *Ficus* sp. [Moraceae], 23.vi.2015, Lihong Dang (**SNUT**); 1♀1♂, Yunnan, Puer, from *Ficusconcinna* [Moraceae], 11.vii.2022, Yanqiao Li (**SNUT**); 4♂, Hainan, Diaoluoshan, from *Ficus* sp. [Moraceae], 5.iv.1984, Weiqiu Zhang (**SCAU**); 1♀1♂, Guangxi, Nanning, from *Ficus* sp. [Moraceae], 3.viii.1985, Weiqiu Zhang (**SCAU**); 1♀1♂, Guangdong, Guangzhou, Fanyu, from *Ficusmicrocarpa* [Moraceae], 17.v.2009, Jiaqian Gao (**SCAU**); 9♀, Yunnan, Simao, 9 females from *Ficus* [Moraceae], 4.viii.2010 (**ANIC**). ***Syntypes***, 1♀1♂ of *annamensis*, Vietnam, Vinh Tan, on *Citrus* sp. [Rutaceae], 7.iii.1925 (**SMF**). ***Lectotype***, 1♀ of *angusticollis*, Indonesia, Java, on Anonaceae sp., 1.ix.1912 (**SMF**). ***Syntype***, 1♀ of *constrictus*, Indonesia, Java, Semarang, in leaf galls of *Ficusbenjamina* [Moraceae], 16.i.2012 (**SMF**). ***Holotype*** and ***paratype***, 1♀1♂ of *bianchii*, Australia, Queensland, Atherton, from *Ficus* leaves [Moraceae], 31.iii.68 (**ANIC**); 5♀1♂, Cairns, from *Ficus* leaves [Moraceae], 12.xi.2007 (**ANIC**); 4♀3♂, Noosa Heads, from *Ficus* leaves [Moraceae], 31.iii.1995 (**ANIC**); 12♀10♂, Brisbane, Mt Cootha, from *Ficusbenjamina* [Moraceae], 13.v.1994 (**ANIC**). 3♀, TIMOR LESTE. Dili, from *Ficusmicrocarpa* leaves [Moraceae], 26.viii.2018 (**ANIC**). 1♀1♂, Malaysia, Kualar Lumpur, from *Ficusbenjamina* leaf gall [Moraceae], 4.x.1973 (**ANIC**). 1♀1♂, the Philippines, Quezon, from *Ficus* [Moraceae], 23.x.2011 (**ANIC**). 7♀3♂, Thailand, Ang Thong, from *Ficus* leaf folds [Moraceae], 9.vi.2018 (**ANIC**). 2♀, Israel, Tel Aviv, from *Ficusmicrocarpa* [Moraceae], i.2014 (**ANIC**).

#### Comments.

This species was described from both sexes and larvae, and the author reported it to be common on *Ficus* leaves at the Botanic Gardens in Buitenzorg [= Bogor], Java. Priesner described *M.annamensis* from two males and one female taken at a coastal locality in Vietnam, and two of these specimens have been studied here. Karny described *M.angusticollis* from an unspecified number of specimens on Annonaceae in Java, without further details. The *M.angusticollis* specimen listed above bears this data as quoted by Priesner (1929a), and the slide is labelled ‘Lectotype’ but that designation apparently was never formally published. Karny (1912) described *M.constrictus* from both sexes taken in leaf galls on *Ficusbenjamina* in northern Java and syntypes have been studied as listed above. These three species have the distinctive fore wing pattern that is here considered to be typical of *M.jordani*. A further slide labelled as *Leptothripsconstrictus* with four specimens (in SMF) has been studied that was collected at the same site as that species. However, those four specimens have fully clear fore wings, are labelled as collected from *Schouteniaovata*, and are here considered to represent *M.schouteniae*. As mentioned above, and judging from the description, *M.apatelus* from India is possibly the same species as *M.jordani*. In descriptions by Ananthakrishnan the lengths of the tergite IX setae and the pronotal epimeral setae are given considerable importance for distinguishing species. The tergite IX setae S1 in *M.jordani* are variable between samples, from slightly longer to slightly shorter than the tube. Similarly, in a single sample of six *M.longisetis* specimens from Java, these setae are usually distinctly longer than the tube but in one of two males they are shorter. Among specimens here identified as *M.jordani* on *Ficus* leaves from various sites, the pronotal posteroangular setae are commonly slightly longer than the epimeral setae. However, in two specimens from *Ficusbenjamina* galls in Malaya they are only half as long as the epimerals. Arbitrary use of differences in lengths of these setae to distinguish species, without consideration of variation within and between populations, may be misleading.

### ﻿ Mesothrips longistylus sp. nov.

Taxon classificationAnimaliaThysanopteraPhlaeothripidae

C0BB0B11-2612-54F8-B3E9-766BF74E918C

https://zoobank.org/1DB00123-0CA9-419D-84C7-D65FFB5B33A7

Figs 2 , 8 , 15–17 , 20 , 24

#### Material examined.

***Holotype***, ♀, China, Sichuan, Ganzi, on leaves of unknown plant, 27.vii. 2013, Jianfeng Wang (**SNUT**); ***paratypes***, 4♀3♂, with same data as holotype (**SNUT**).

**Description. Holotype. *Female macroptera*.** Body brown; all femora and tibiae brown, all tarsi yellowish brown, slightly lighter than tibiae; antennal segments I and II brown, III clear yellow, IV–VI yellow but shaded on apical half, VII–VIII brown (Fig. 8); major setae pale; fore wing pale.

***Head*.** Head ~ 1.4 × as long as wide (Fig. 2), constricted at base; postocular setae slightly blunt, shorter than eyes (Fig. 2); eyes equal in length ventrally and dorsally; maxillary stylets parallel medially with transverse maxillary bridge, elongate and at full retraction extending to postocular setae (Fig. 2); mouth cone long, reaching to ferna. Antennal segments broad (Fig. 8), segment III ~ 2.1 × as long as apical width; III with three sense cones, IV with four major sense cones, VIII broadly connected with VII.

***Thorax*.** Pronotum with five pairs of well-developed setae, am, aa and ml blunt at apex (Fig. 2), epim and pa longer and equal in length, slightly expanded at apex; surface almost smooth, with weak sculpture near margins. All legs slender, fore tarsal tooth tiny, visible only when tarsi rotated. Fore wing with three sub-basal setae arising in straight line (Fig. 20), S1 and S2 equal in length, slightly expanded at apex, S3 longest, acute at apex, with 9–12 duplicated cilia. Mesonotum transversely reticulate, lateral setae well-developed, blunt (Fig. 15). Metanotum longitudinally reticulate, major setae slender and acute (Fig. 15). Mesopresternum with paired lateral triangles, metathoracic sternopleural sutures absent.

***Abdomen*.** Pelta broadly triangular, weakly reticulate, with pair of CPS (Fig. 16); tergites II–VII with two pairs of major wing retaining setae, one pair of accessory sigmoid setae located anterior to first pair (Fig. 17); tergite II with eight pairs of lateral setae; tergite IX setae S1 and S2 longer than tube, acute at apex (Fig. 17), S3 approx. as long as tube, acute at apex; tube shorter than head, anal setae approx. as long as tube.

***Measurements*** (holotype female in μm). Body length 2600. Head length (maximum width) 275 (195); distance between maxillary stylets (across bridge) 50; postocular setae length 70; antennal segments I–VIII length (width): 45 (35), 55 (30), 75 (35), 75 (40), 70 (30), 65 (30), 60 (25), 35 (20); sense cone on III length 15. Pronotum length (width) 170 (275); am 45, aa 35, ml ?, epim 90, pa 90. Fore wing length 1140; sub-basal setae S1 60, S2 85, S3 120. Tergite IX setae S1 185, S2 190, S3 140; tube length 170, basal width 75, apical width 50; anal setae length 170.

***Male macroptera*.** Similar to female in colour and sculpture; postocular setae slightly shorter than eyes, slightly blunt at apex; fore tarsal tooth scarcely visible; abdominal tergite IX setae S2 small and pointed (Fig. 24); sternite VIII without pore plate.

**Figures 26–31.**
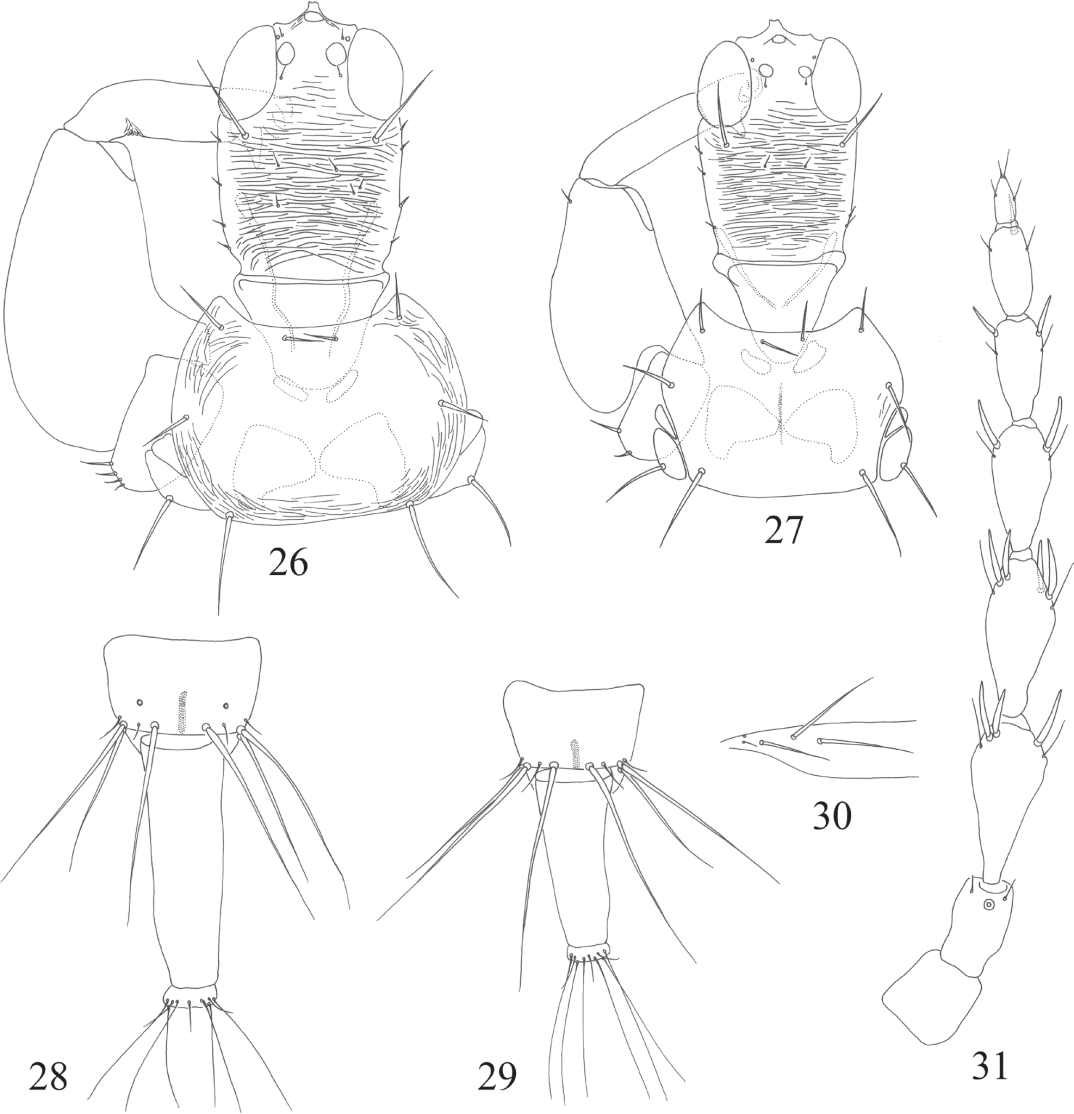
*Mesothrips* spp. Head, pronotum and foreleg (**26–27**) **26***M.pyctes***27***M.vitripennis* (type specimens of *elaeocarpi*); tergites IX–X (**28–29**) **28***M.pyctes***29***M.vitripennis* (type specimens of *elaeocarpi*); *M.pyctes* (**30–31**) **30** sub-basal setae of forewing **31** antennae.

***Measurements*** (paratype male in μm). Body length 2240. Head length (maximum width) 270 (200); postocular setae length 90. Pronotum length (width) 170 (270); am 53, aa 28, ml 75, epim 75, pa 85. Tergite IX setae S1 180, S2 20, S3 180; tube length 165; anal setae length 175.

#### Etymology.

The species epithet refers to the elongate and parallel maxillary stylets.

#### Comments.

The elongate and parallel maxillary stylets of this new species are unique in *Mesothrips*, most of which species have the stylets wide apart and arranged in a V-shape.

### ﻿ Mesothrips pyctes

Taxon classificationAnimaliaThysanopteraPhlaeothripidae

Karny

0C837814-77EA-5773-8403-64890C6FD331

Figs 26 , 28 , 30 , 31

Mesothrips pyctes Karny, 1916: 191.

#### Material examined.

***Syntypes***, 1♀1♂, Indonesia, Java, on *Ficus* sp. [Moraceae], 1.iii.1914, Docters van Leeuwen (**SMF**).

#### Comments.

This species was described from an unspecified number of adults taken in Java, Indonesia, from leaf galls on a species of *Eugenia* [Myrtaceae]. No further studies were published on this species until Wang (2002) listed it from Taiwan, presumably based only on the key to species by Ananthakrishnan (1976). Fortunately, one female and one male labelled as syntypes, but labelled as taken from *Ficus* sp., were checked during studies in 2013 (Dang et al. 2014). *Mesothrips* species usually have setae on tergite IX as long as or longer than tube, but these syntypes have these setae shorter than the tube (Fig. 28), a condition that also occurs in *M.claripennis*. However, it seems that they can be distinguished by the length of the anal setae (Fig. 28), as indicated in the key.

### ﻿ Mesothrips vernicia sp. nov.

Taxon classificationAnimaliaThysanopteraPhlaeothripidae

1052938D-574C-5A5B-82CE-BFCBA9D7672A

https://zoobank.org/4DEE6509-DB4E-4A71-8D2A-E233B77B6E4A

Figs 4 , 9 , 12 , 21 , 25

#### Material examined.

***Holotype***, ♀, China, Sichuan, Guangyuan, on leaves of *Vernicia* sp. [Euphorbiaceae], 07.viii.2018, Lihong Dang, Yang Hu and Danle Xie (**SNUT**); ***paratypes***, 2♀3♂, with same data as holotype (**SNUT**); ♀, Hubei, Luotian, on leaves of unknown tree, 03.vii.2014, Lixin Su (**SNUT**).

#### Description.

**Holotype. *Female macroptera*.** Body brown; all femora and tibiae brown, fore tarsi clear yellow, mid and hind tarsi brownish, slightly lighter than tibiae; antennal segments I and II brown, III–VI uniformly yellow, VII yellow on basal 2/3, and light brown on apical 1/3, VIII lightly brown (Fig. 9); major setae pale; fore wing slightly greyish.

***Head*.** Head ~ 1.5 × as long as wide (Fig. 4), constricted at base; postocular setae blunt, ~ 1/2 length of eyes (Fig. 4); eyes longer ventrally than dorsally; maxillary stylets V-shaped, retracted to median of head; mouth cone long, reaching to ferna. Antennae slender, segment III ~ 3.2 × as long as apical width; III with two sense cones, IV with four major sense cones, VIII broadly connected with VII (Fig. 9).

***Thorax*.** Pronotum with five pairs of blunt setae, am, aa and pa equal in length (Fig. 4), epim and pa longer and equal in length; surface almost smooth, with weak sculpture near margins. All legs slender, fore tarsal tooth absent (Fig. 12). Fore wing with three blunt sub-basal setae arising in straight line, S1 and S2 equal in length, S3 longest, with seven duplicated cilia. Mesonotum transversely reticulate, lateral setae well-developed, blunt. Metanotum longitudinally reticulate, major setae slender and acute. Mesopresternum with paired lateral triangles, metathoracic sternopleural sutures absent.

***Abdomen*.** Pelta broadly triangular, weakly reticulate, with pair of CPS; tergites II–VII with two major pairs of wing retaining setae, one pair of accessory sigmoid setae located anterior to first pair (Fig. 21); tergite II with four pairs of lateral setae; tergite IX setae S1 and S2 longer than tube, acute at apex, S3 approx. as long as tube, acute at apex; tube shorter than head, anal setae approx. as long as tube.

***Measurements*** (holotype female in μm). Body length 2620. Head length (maximum width) 260 (175); distance between maxillary stylets (across bridge) 120; eye length dorsally 90, ventrally 120; postocular setae length 45; antennal segments I–VIII length (width): 40 (30), 50 (35), 80 (25), 80 (30), 75 (30), 65 (20), 50 (20), 25 (15); sense cone on III length 20. Pronotum length (width) 170 (240); am ?, aa 35, ml 25, epim 70, pa 55. Fore wing length 1000; sub-basal setae S1 45, S2 50, S3 95. Tergite IX setae S1 195, S2 185, S3 150; tube length 150, basal width 75, apical width 45; anal setae length 170.

***Male macroptera*.** Similar to female in colour and sculpture; antennal segment VII largely brown; fore tarsal tooth absent; abdominal tergite IX setae S2 small and pointed (Fig. 25); sternite VIII without pore plate.

***Measurements*** (paratype male in μm). Body length 2100. Head length (maximum width) 240 (145); eye length dorsally 85, ventrally 85; postocular setae length 45. Pronotum length (width) 135 (185); am 25, aa 30, ml 20, epim 45, pa 25. Tergite IX setae S1 180, S2 15, S3 180; tube length 145; anal setae length 175.

#### Etymology.

The species epithet refers to the genus name of the host plant.

**Comments.** As indicated above, the relationships of this species remain far from clear. It is similar in structure to the other two new species but differs sharply from the other *Mesothrips* species in lacking a prominent fore tarsal tooth (Fig. 12), and in the presence of only two sense cones on the third antennal segment (Fig. 9).

### ﻿ Mesothrips vitripennis

Taxon classificationAnimaliaThysanopteraPhlaeothripidae

Karny

FE711CA7-C85D-5603-9723-4B5C291F2BB7

Figs 6 , 11 , 19 , 27 , 29

Mesothrips vitripennis Karny, 1922: 149.
Mesothrips moundi Ananthakrishnan, 1976: 195. Syn. nov.
Mesothrips elaeocarpi Ananthakrishnan, 1976: 192. Syn. nov.

#### Material examined.

1♂, China, Guangxi, Chongzuo, from tree leaves, 25.vii.2021, Xia Wang (**SNUT**); ***paratype***, 1♀ of *moundi*, Hongkong, Tai Ling, from leaves of *Bischofiatrifoliata* [Phyllanthaceae], 5.v.1966 (**ANIC**). 1♀1♂, Vietnam, Kha-Tin, from leaf gall on *Aporosaleptostachya* [Phyllanthaceae], 20.xi.1920, Docters v. Leeuwen-No. 86 (**SMF**). ***Paratype***, 1♀ of *elaeocarpi*, Indonesia, Java, Bogor, from leaf gall of *Elaeocarpusstipularis* [Elaeocarpaceae], 28.v.1925 (**SMF**). 1♀, INDIA, Courtallam, on *Mallotus* sp. [Euphorbiaceae], 26.vii.1967, identified by Ananthakrishnan (**SMF**).

#### Comments.

The original description states that this species was collected at Saigon from leaf galls on *Aporosa* on 19.x.1920, whereas the specimens studied here were labelled by Karny from a slightly different locality, collected 10.xi.1920 from leaf galls on *Aporosaleptostachya*. From a plant in the same family, Ananthakrishnan described *M.moundi* (Figs 6, 11) from 17 females and 3 males taken in Hong Kong, and a paratype female is listed below as studied here. This specimen cannot be distinguished as a separate species from the *M.vitripennis* specimens. The head of *M.vitripennis* is rather short, the tube is short and approx. as long as the head width, tergite IX setae S1 are longer than the tube and the fore wings are pale. The new synonymy of *M.elaeocarpi* is based on the paratype female listed below, also one female from India identified by Ananthakrishnan.

## Supplementary Material

XML Treatment for Mesothrips

XML Treatment for Mesothrips claripennis

XML Treatment for Mesothrips jianfengi

XML Treatment for Mesothrips jordani

XML Treatment for Mesothrips longistylus

XML Treatment for Mesothrips pyctes

XML Treatment for Mesothrips vernicia

XML Treatment for Mesothrips vitripennis

## ﻿Additional information

### Author ORCIDs

Lihong Dang https://orcid.org/0000-0002-7571-8426

Xiaoli Tong https://orcid.org/0000-0003-1731-229X

Laurence A. Mound https://orcid.org/0000-0002-6019-4762

### Data availability

All of the data that support the findings of this study are available in the main text.
